# Effect of *Macleaya cordata* and *Magnolia officinalis* plant extracts on oxidative stress control in lambs fed a high-concentrate diet

**DOI:** 10.5713/ajas.19.0050

**Published:** 2019-07-01

**Authors:** Tiago Ronimar Ferreira Lima, Sarita Bonagurio Gallo, Alessandra Fernandes Rosa, Saulo da Luz e Silva, Thais Brochado, Helena Viel Alves Bezerra, Soraia Marques Putrino, Marcela Buosi Martins, Paulo Roberto Leme

**Affiliations:** 1 Department of Animal Science, University of São Paulo, Duque de Caxias Norte ave. Pirassununga, SP 13635-900, Brazil; 2 Neovia Group, João Augusto Cirelli St., Descalvado, SP 3690-000, Brazil

**Keywords:** Sheep, *Macleaya cordata*, *Magnolia officinalis*, Selenium, Vitamin E

## Abstract

**Objective:**

The objective of this experiment was to compare conventional antioxidants and plant extracts for oxidative stress control in lambs fed a high-concentrate diet.

**Methods:**

Forty-eight male Dorper×Santa Ines lambs with an initial weight of 20±1.49 kg and 60 days of age, were used to evaluate the effects of feeding a combination of *Macleaya cordata* and *Magnolia officinalis* plant extracts (0 vs 320 mg/kg dry matter [DM]) in combination with selenium+vitamin E (0 vs 100 IU/kg DM of vitamin E and 0.1 mg/kg DM of selenium) in a completely randomized block design in a 2×2 factorial arrangement. The animals were housed in individual pens and received a high-concentrate diet consisting of 80% whole corn and 20% protein pellet for 60 days. The animals were weighed at the beginning of the experiment and every 14 days for performance monitoring. Three blood samplings were performed during the experimental period for the evaluation of oxidative and protein parameters.

**Results:**

The treatments with vitamin E and selenium as additives had a positive influence on final weight, daily weight gain, carcass weight, and selenium content in longissimus muscle (p = 0.01). Plant extracts tended to improve final weight (p = 0.064) and daily weight gain (p = 0.059), showing similar effect as selenium and vitamin E. There was no effect of treatment on blood proteins, indicating that the animals were healthy throughout the experiment.

**Conclusion:**

The use of plant extracts had a similar effect as the addition of selenium and vitamin E, with dietary inclusion of additives resulting in better performance of lambs but both supplements did not have strong influence on oxidative stress.

## INTRODUCTION

In intensive meat production systems, the animals are challenged nutritionally by the use of very low-fiber diets, especially during the feedlot finishing phase [1]. The use of high-concentrate diets permits to increase sheep meat production, ensuring a shorter time to slaughter because of high feed efficiency and better carcass uniformity [2]. Despite benefits for animal productivity, these diets can affect metabolism and this interference can result in the release of diverse compounds into the blood circulation, influencing redox homeostasis of the organism. To prevent oxidative stress in the animal’s body, antioxidants such as selenium (Se) and vitamin E have been used to combat free radicals that are harmful when present above normal levels [3]. In a study in which acute mastitis was experimentally induced in cows, the administration of vitamin E and Se resulted in an increase in antioxidant capacity. Dietary administration of Se alone or in combination with vitamin E increases the defense capacity of blood neutrophils against bacterial infections and can enhance red blood cell resistance to oxidative stress [4,5].

Some active compounds extracted from plants can be used as an alternative for oxidative stress control because of their capacity to neutralize free radicals. These compounds include honokiol and its isomer magnolol and sanguinarine extracted from the plants *Magnolia officinalis* and *Macleaya cordata*, respectively. In addition to their antioxidant effects, the active compounds have anti-inflammatory, antifungal, and antimicrobial properties, among others [6-8]. The honokiol molecule possesses two phenol groups that confer properties similar to those of vitamin E. Inducing cell membrane damage by toxicity in cultured rat cerebellar granule cells, a study reported that honokiol and magnolol reversed the induced mitochondrial dysfunction and vitamin E also provided protection against this damage, suggesting neuroprotective activity of these compounds due to their antioxidant properties [9].

Since honokiol and magnolol possess strong antioxidant activity, the objective of this study was to evaluate the effects of *Magnolia officinalis* and *Macleaya cordata* extracts on oxidative stress control in lambs fed a high-concentrate diet, as well as their influence on animal performance and carcass and meat quality traits.

## MATERIALS AND METHODS

### Animal care

The experimental procedure was approved by the Ethics Committee on Animal Use of the Faculty of Animal Science and Food Engineering, University of São Paulo (approval number 9864230215).

### Animals, facilities and treatments

Forty-eight uncastrated male Dorper×Santa Ines lambs (20± 1.49 kg of body weight [BW] and 60 days of age) were used in a completely randomized block (initial BW) design in a 2×2 factorial arrangement to evaluate the effect of supplying plant extract (0 or 320 mg/kg of dry matter [DM]) or Se+vitamin E (0 or 0.1 mg selenium/kg of DM and 100 IU vitamin E/kg of DM) on performance, rumen health, oxidative stress, and carcass and meat quality traits.

The lambs were maintained in individual pens (1.1×1.2 m) with a wood pallet floor equipped with troughs and drinking fountains. The animals were allowed to adapt to the diets and facilities for 7 days, receiving *ad libitum*, twice daily, a standard diet of 100% concentrate consisting of whole corn and protein pellet at a proportion of 80:20 (Table 1) without additives. After this period, the animals were randomly assigned to the treatments.

The amount of feed supplied and leftovers were recorded daily. The samples were duly conditioned in plastic bags for analysis of DM, crude protein, non-protein nitrogen, acid detergent fiber, and neutral detergent fiber, according to AOAC [10]. Total digestible nutrients were estimated according to Weiss et al [11].

The Se content in the pellets of the treatments was analyzed by fluorimetry [12], with concentrations of 0.078, 0.068, 1.025, and 0.993 mg/kg DM, respectively. The concentration in the whole corn was 0.04 mg Se/kg DM. Thus, the final diets of the control, ExP, SeE, and SeE+ExP treatments contained 0.047, 0.046, 0.237, and 0.231 mg Se/kg DM, respectively. The Se requirement for sheep of this category is 0.26 mg/kg DM [13]. The diets of the control and ExP treatments contained 0.39 mg vitamin E/kg of feed and the SeE and SeE+ExP treatments contained 18.59 mg/kg. The functional components of plant extract *Magnolia officinalis* is honokiol and magnolol (neo-lignans) and of plant extract *Macleaya cordata* is sanguinarine (alcaloids). The dose of ExP was indicated by the producers of the additive.

The chemical composition of the mineral protein pellet and the nutrients of the feeds used in this study are shown in Table 1. Two animals showed health problems during the text and were dropped from the trial.

### Sample collection and laboratory analysis

Daily dry matter intake (DMI) was calculated as the quantity of DM of the diet supplied minus the quantity of DM of the leftovers. Feed efficiency of the animals was obtained as the ratio between weight gain and DMI. For the monitoring of average daily gain (ADG), the animals were weighed every 14 days in the morning, without prior fasting to avoid possible acidosis.

For oxidative stress analysis, blood samples were collected at days 25, 32, and 46 of the experiment to evaluate enzyme activity and the oxidant/antioxidant balance. The blood samples were collected into vacutainer tubes, one with ethylenediaminetetraacetic acid (EDTA) for the separation of plasma and one without EDTA for the separation of serum. The levels of glutathione peroxidase (GPx), thiobarbituric acid reactive substances, and superoxide dismutase (SOD) were evaluated in plasma using commercial kits (Cell Biolabs, Inc., San Diego, CA, USA). The enzymatic activity was evaluated in the plasma because of its greater capacity of conservation of metabolites, due to the non-occurrence of a coagulation cascade, allowing the enzymes not to be retained in the platelet aggregate, promoting better homogeneity of the metabolites in detriment of the serum. The serum was separated for the measurement of serum proteins, according to the methodology proposed by Laemmli [14].

### Serum protein electrophoresis

Serum protein electrophoresis was performed using the previously collected serum samples according to the technique described by Laemmli [14], with modifications, in a vertical electrophoresis system. The samples were prepared using 10 μL blood serum diluted in 30 μL phosphate-buffered saline (PBS) and 20 μL sample dilution buffer (0.5 M Tris-HCl, pH 6.8, 10% glycerol, 10% (w/v) sodium dodecyl sulfate, 5% 2-mercaptoethanol, and 1% bromophenol blue) and heated to 100°C for 10 min. A 5-μL aliquot of the samples was loaded into each well of the gel. The gel was run at 20 mA for 120 min.

After separation, the gel was stained with 0.2% Coomassie blue solution for 2 h and excess dye was removed with decolorizing solution until the fractions became clear. For the determination of molecular weight and concentration of the protein fractions, the gels were scanned with a computerized densitometer. Molecular weight markers of 200, 116, 97, 66, 55, 45, 36, 29, and 20 kDa, as well as purified albumin, haptoglobin, ceruloplasmin, transferrin, and immunoglobulin G (IgG) were used for protein identification. Reference curves constructed from the standard marker were used for densitometric evaluation of the protein bands.

### Slaughter, sample collection, and measurement of carcass traits

After 60 days of feeding, the animals were slaughtered after fasting from solids for 18 h at the slaughterhouse of the University of São Paulo, located 200 m from the experimental site. Slaughter was performed following the humane procedures established by the Brazilian legislation and the animals were stunned with a penetrating captive bolt gun and bled through the jugular vein and carotid artery. The carcasses were then skinned, eviscerated, washed, identified, weighed, and stored in a cold storage room (0°C to 2°C) for 24 h. After this period, the carcasses were again weighed for the determination of cold carcass weight, pH, and temperature measured in longissimus muscle in the region of the 12th rib using a digital pH meter with a penetration probe (model HI8314, Hanna Instruments, Woonsocket, RI, USA).

The left half-carcass was cut between the 12th and 13th rib for the measurement of loin eye area (LEA) with a specific 1-cm^2^ transparent grid and of subcutaneous fat thickness (SFT) using a ruler with millimeter graduation.

### Physical analysis of meat

For the analysis of shear force (tenderness) and cooking losses, approximately 2.5 cm-thick steaks were removed from the longissimus muscle between the 12th and 13th rib, vacuum packed, and immediately frozen for subsequent analysis. On the day prior to tenderness evaluation, the samples were thawed in a refrigerator (2°C to 5°C), removed from the packaging, and weighed individually for the determination of initial weight. Next, a thermometer was inserted into the geometric center of each steak and the samples were heated in an industrial electrical oven (Model F130/L, Fornos Elétricos Flecha de Ouro Ind. e Com. Ltda., São Paulo, SP, Brazil) to 170°C until the internal temperature reached 40°C. The steaks were then turned and cooked until an internal temperature of 71°C was reached. The samples were cooled at room temperature (22°C), weighed again, wrapped in plastic film, and stored in the refrigerator (4°C to 6°C) until the next day.

Three cylinders (1.27 cm in diameter) were removed from each sample parallel to the muscle fiber orientation and sheared in a TMS-PRO texture analyzer (Food Technology Corporation, Sterling, VA, USA) for the determination of shear force. The shear force of each sample was obtained as the mean of three replicates and is expressed as newton (N). Cooking losses were calculated as the difference between the initial and final weight of the sample, divided by the initial weight and multiplied by 100 [15].

The content of Se in muscle was determined by a fluorometric method [12] using the bovine liver standard (1577c) from the National Institute of Standards and Technology (NIST, Gaithersburg, MD, USA) as quality control.

### Analysis of rumen morphology

After evisceration, the rumen was separated from the other compartments, opened, and washed under running water for the determination of ruminitis score. The ruminal papillae were classified visually according to the presence of lesions on a scale from 0 to 10, where each score indicates 10% of compromised rumen. Any classification above zero was defined as the occurrence of ruminitis [16].

For assessment of papilla morphology, a fragment of approximately 3 cm^2^ was collected from the cranial sac of each rumen and immediately stored in flasks containing PBS to preserve the biological characteristics. The number of papillae/cm^2^ was estimated by counting the papillae present in the fragments by three people, considering the average of the three counts. Next, the fragments and twelve papillae were sectioned, scanned and with software UTHSCSA Image Tool [17] the mean papillary area, percent papillary area, and total surface area for absorption per cm^2^ of rumen wall were determinated.

### Statistical analysis

The data were analyzed by analysis of variance using the Mixed procedure of the SAS 9.3 software (SAS Institute, Inc., Cary, NC, USA), considering the fixed effects of plant extract (0 or 320 mg/kg DM), Se+vitamin E (0 or 0.1 mg selenium/kg DM and 100 IU vitamin E/kg DM) and their interaction as fixed effects and the block as random effect. When a significant effect of the main factors was observed, treatment means were compared using the LSMEANS option adjusted by the Tukey test. The animal (pen) was considered the experimental unit. Due to the loss of two experimental units (two lambs with urolithiasis), the data of 46 animals were statistically evaluated.

## RESULTS

### Performance and carcass and meat quality traits

There was no interaction between any of the performance and carcass traits evaluated. An increase in ADG (p = 0.045) was observed with the inclusion of SeE (Table 2). The DMI was proportional to the weight of the animals, without differences in feed efficiency or interaction between treatments. A higher final weight (p = 0.014) and carcass weight (p = 0.011) were observed for animals receiving the SeE treatment. The treatments did not influence LEA, SFT, pH, or carcass temperature at 1 h or 24 h after slaughter.

No difference was found in shear force, which was below 16 N for all treatments. There was also no difference in cooking loss among treatments, with mean losses ranging from 23.2% to 24.6% (Table 3).

For the treatment with the addition of Se, as expected, the concentration of this element in muscle was higher (p = 0.0001) and treatment ExP did not affect its concentration.

### Rumen morphology parameters

Analysis of rumen morphology parameters (Table 4) showed no difference or interaction between treatments for ruminitis score, which did not exceed 1 (10% of the rumen with lesions). There were no differences in the number of papillae, surface area for absorption, or mean papillary area. The percent papillary area was greater for treatment ExP (p = 0.0459).

### Oxidative stress

**Figure 1:**
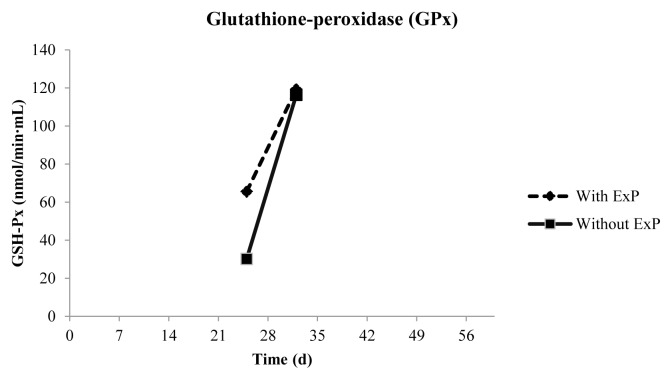
Glutathione-peroxidase activity in plasma of Dorper×Santa Ines lambs receiving a high-concentrate diet with different antioxidant additives at first and second sampling.

Regarding the oxidative stress parameters, there was no difference in malondialdehyde levels (MDA/μM) among treatments or interaction between factors (Table 5). In animals receiving treatment Se, GPx activity in plasma increased from the first to the second sampling (p<0.0001) (Figure 1), and an effect of time on the activity of this enzyme was also observed (p< 0.0001).

**Figure 2:**
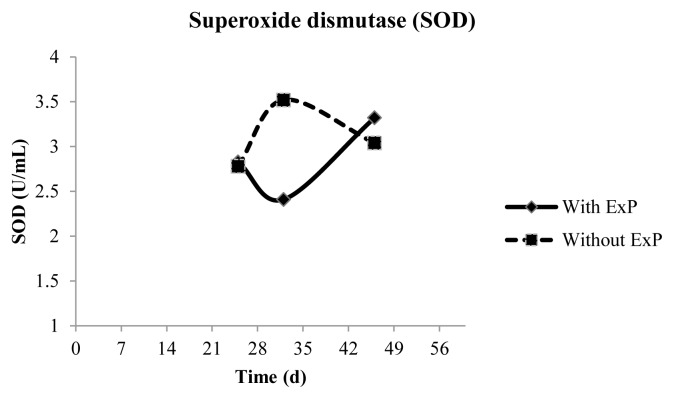
Higher superoxide dismutase activity in plasma of in Dorper×Santa Ines lambs receiving a high-concentrate diet with different antioxidant additives at d 32.

There was an interaction between time and treatment (p = 0.0172) for SOD activity, in which animals that did not receive ExP exhibited higher enzymatic activity than treated at d 32. The activity of this enzyme was the same across treatments in the last sampling (Figure 2).

### Blood proteins

The concentration of haptoglobin (Table 6) in serum was lower (p = 0.035) for animals treated with ExP, but there was no interaction or effect of treatment SeE. The levels of ceruloplasmin, albumin, IgG heavy and light chain, transferrin or glucopolysaccharide, which are important for good physiological function, did not differ among treatments.

## DISCUSSION

In the present study, no difference was observed in DMI expressed as % BW, while ADG was increased by SeE and tended to be increased by ExP, and there was no interaction. In a study comparing the supplementation of lambs with 0.15 and 0.30 ppm Se, higher ADG was observed for supplemented animals compared to control [18].

The hot carcass yields of this experiment were similar than those observed in the literature, in which studies reported a hot carcass yield of 48.9% for Santa Ines lambs fed a high proportion of concentrate and of 50.6% for Santa Ines lambs fed diets containing 90% concentrate and slaughtered at a mean weight of 39.5 kg [19,20]. This variation can be explained by the fact that crossing Santa Ines with Dorper breed incresed hot carcass yield, in addition to slaughter weight which ranged from 39 to 45 kg.

There was no difference in SFT among treatments despite the high energy content of the diet and its influence on fat deposition, since all treatments contained the same amount of energy and only differed in terms of the presence or absence of additives.

The tenderness of sheep meat can be classified into four classes: tender when shear force is less than 2.27 kg (22.26 N), medium when shear force ranges from 2.28 to 3.63 kg (22.35 to 35.59 N), tough when shear force ranges from 3.64 to 5.44 kg (35.69 to 53.35 N), and very tough when shear force is greater than 5.44 kg (53.35 N) [20]. Thus, the meat of the lambs of this experiment can be classified as tender.

The amount of Se supplied to the animals was based on requirements and not on supranutritional supplementation. The difference in average Se concentrations compared to other experiments that used mineral supplementation was therefore expected. Higher Se concentrations were observed for the treatments with Se addition compared to those without addition, demonstrating the extra deposition of the mineral in muscle. Higher amounts of Se were also expected for the ExP treatment as the Se that would be used for selenoproteins to combat oxidative stress would be directed to the muscle, with the plant extract additive playing the role of protection against oxidative damage, but this did not occur in this study.

A ruminitis score less than 1 (10% of rumen lesions) was obtained for all treatments. The development of ruminal papillae is related to the supply of non-fibrous carbohydrates, which act on the production of short-chain fatty acids.

The level of total protein is proportional to the amount supplied in the diet, in addition to providing information about protein and hepatic metabolism. The present results agree with the mean values for lambs, which range from 6.0 to 7.9 g/dL [21,22]. Acute-phase proteins are related to physiological and metabolic alterations resulting from inflammatory processes. This class of proteins can be divided into positive acute-phase proteins whose plasma concentration increases in response to inflammation. These proteins include ceruloplasmin, fibrinogen, C-reactive protein, antitrypsin and haptoglobin, which exert a significant influence on animal performance. On the other hand, the concentration of negative acute-phase proteins such as albumin, transferrin and pre-albumin decreases in response to inflammation [22].

Haptoglobin is considered a biomarker of inflammatory and infectious processes in humans and animals which, together with ceruloplasmin, is six times more sensitive in detecting inflammation than fibrinogen, total leukocyte count or segmented and banded neutrophil count. This higher sensitivity is due to the significant increase in the levels of this protein in the first hours after exposure to a pathogen before the manifestation of clinical signs [23,24]. Within this context, haptoglobin might be an important biomarker for health monitoring of slaughter animals since healthy animals have low levels of this protein, while sick animals exhibit elevated levels [25]. In the present experiment, the animals treated with ExP had lower serum haptoglobin concentrations, indicating that ExP lowers systemic inflammation.

Oxidative stress can be influenced by several factors, including physical, chemical, physiological and environmental factors, which can trigger or aggravate clinical conditions in animals. In a study evaluating antioxidant status and oxidative stress in sheep with parasitemia, higher MDA values were observed in infected animals compared to control animals (68.42 vs 28.47 nmol/g hemoglobin) [26].

Dietary Se addition is positively correlated with circulating GPx activity since this mineral is a central part of this protein. The addition of Se and vitamin E increased the enzymatic activity of GPx. This enzyme reduces hydrogen peroxide and other peroxides to water or alcohol. The GPx is a selenoprotein that contains Se in its active site, which is a known antioxidant nutrient. The levels of GPx found in healthy lambs were 132.3 IU/g hemoglobin, while lambs with nutritional muscular dystrophy in cardiac and skeletal muscle had activities of 21.9 and 45.7 IU/g hemoglobin, respectively [27]. In contrast, lower GPx values were observed in animals infected with *Babesia ovis*. In this case, the greater the infestation, the lower the GPx values, with an enzymatic activity of 39.19 IU/mg hemoglobin [26]. Selenium is a structural part of the GPx enzyme and its greater availability positively influences the synthesis of new enzymes. Although the stress factor differs among studies, it is clear that the increase in the intensity of the stressor has a negative impact on GPx activity [28].

Superoxide possesses an irrelevant oxidizing function since, unlike other free radicals, it is present in the inactive form. However, superoxide becomes important because it participates in the formation of hydrogen peroxide by a dismutase reaction of two superoxide molecules, which is catalyzed by SOD. If not eliminated, peroxidases and catalases transform peroxides into hydroxyl radicals, which are highly damaging to the organism. The increased activity of SOD may be related to higher hydrogen peroxide production [29].

In conclusion, the use of plant extract or selenium and vitamin E for lambs fed a high-concentrate diet did not interfere with most of the characteristics studied. However, the animals exhibited better performance than those that received the diet without additives. Regarding performance traits and ruminitis indicators, the effect of supplementation with plant extract was similar to that of supplementation with Se+vitamin E. However, both supplements did not have strong influence on oxidative stress.

## Figures and Tables

**Table 1:** Ingredients and composition of the pellet

Items	
Pellet ingredients (% dry matter)	
Wheat bran	35
Corn germ	5.32
Fat rice bran	10
Soybean meal	21.14
Cottonseed meal	10
Microgranulated dicalcium phosphate	0.65
Gray calcareous1)	5.33
Sodium chloride	0.19
Sodium bicarbonate	1.5
Urea	5
Binder2)	0.5
Kaolin3)	3.88
Mineral premix4)	1.5
Composition of the diets containing 80% corn and 20% pellet (% dry matter)	
Crude protein	15.45
Total digestible nutrients5)	81.83
Ether extract	4.94
Non-protein nitrogen	1.04
Minerals	4.3
Acid detergent fiber	3.77
Neutral detergent fiber	12.26
Calcium	0.53
Phosphorus	0.43
Sodium	0.16
Sulfur	0.07
Iron (mg/kg)	32
Manganese (mg/kg)	40.8
Zinc (mg/kg)	77.6
Cobalt (mg/kg)	0.43

1) Source of calcium.

2) Pellet binding agent.

3) Vehicle, has no nutritional value.

4) The mineral premix of the control treatment had 54.142% lime, 0.889% iron sulfate, 2.000% manganese monoxide, 5.715% zinc sulfate, 0.400% chromium chelate, 0.167% A vitamim, 0.054% D_3_ vitamin and 37.522% inert vehicle (kaolin). In the ExP treatment the vehicle was replaced with 10.8% of plant extract, in the SeE treatment replaced with 0.0244% sodium selenite and 0.91% vitamin E and in the SeE+ExP treatment replaced with the same percentages of the SeE and ExP treatments.

5) Estimated according to Weiss et al [11].

**Table 2:** Performance and carcass data of Dorper×Santa Ines lambs receiving a high-concentrate diet with different antioxidant additives

Items	SeE		ExP		SEM	Pr>F		
		
	With	Without	With	Without		SeE	ExP	SeE×ExP
Initial BW (kg)	21.5	20.1	21.1	20.56	1.99	0.1076	0.5247	0.7490
Final BW (kg)	44.6	41.0	44.1	41.43	2.47	0.0149	0.0641	0.3646
ADG (kg)	0.390	0.352	0.389	0.353	0.01	0.0451	0.0596	0.3768
DM intake (kg/d)	1.5	1.4	1.5	1.4	0.07	0.0272	0.4127	0.9725
DM intake (% BW)	3.4	3.3	3.3	3.4	0.11	0.3034	0.3391	0.2958
Feed efficiency (g/kg DMI)	268.4	273	278.5	0.262	0.01	0.6353	0.1102	0.2163
Carcass weight (kg)	21.2	19.2	20.5	20.0	1.09	0.0113	0.5246	0.8304
Carcass yield (%)	48.81	49.5	49.01	49.3	0.34	0.1539	0.5487	0.1373
Loin eye area (cm^2^)	14.4	13.3	13.8	14.0	0.50	0.1275	0.6976	0.2221
Subcutaneous fat thickness (mm)	3.5	3.4	3.4	3.5	0.17	0.5674	0.8169	0.9514
pH 24 h	5.9	5.8	5.8	5.9	0.08	0.8404	0.5253	0.1503
Temperature 24 h	5.8	5.5	5.5	5.8	0.25	0.4916	0.4916	0.4916

SeE, vitamin E+selenium; ExP, plant extracts; SEM, standard error of the mean; BW, body weight; ADG, average daily gain; DM, dry matter; DMI, dry matter intake.

**Table 3:** Shear force, cooking loss and selenium content in meat of Dorper×Santa Ines lambs receiving a high-concentrate diet with different antioxidant additives

Items	SeE		ExP		SEM	Pr>F		
		
	With	Without	With	Without		SeE	ExP	SeE×ExP
Shear force (N)	16.5	15.0	15.1	16.4	0.89	0.9636	0.9824	0.165
Cooking loss (%)	24.6	23.2	23.3	24.5	0.8525	0.2568	0.3531	0.1508
Se (mg/kg)	0.057	0.024	0.041	0.040	0.0021	<0.0001	0.6609	0.1927

SeE, vitamin E+selenium; ExP, plant extracts; SEM, standard error of the mean.

**Table 4:** Average ruminitis score and ruminal wall morphology of Dorper×Santa Ines lambs receiving a high-concentrate diet with different antioxidant additives

Items	SeE		ExP		SEM	Pr>F		
		
	With	Without	With	Without		SeE	ExP	SeE×ExP
Ruminitis score	0.4	0.5	0.3	0.5	0.17	0.8063	0.4479	0.8063
Absorption surface/cm^2^ of wall (cm^2^)	16.4	15.6	17.0	15.0	0.93	0.5024	0.2318	0.6035
Papilla number/cm^2^ of wall	28.1	27.4	29.9	25.5	1.57	0.7554	0.0571	0.7066
Papillary area (% absorption surface)	95.1	94.6	95.1	92.0	0.32	0.0582	0.0459	0.5381
Papillary area (cm^2^)	0.42	0.37	0.52	0.40	0.04	0.2157	0.1893	0.3574

SeE, vitamin E+selenium; ExP, plant extracts; SEM, standard error of the mean.

**Table 5:** Oxidative stress parameters in the plasma of lambs receiving a high-concentrate diet with different antioxidant additives

Items	SeE		ExP		SEM	p-value				
		
	With	Without	With	Without		SeE	ExP	Days	SeE×Days	ExP×Days
TBARS (MDA/μM)	21.78	13.81	24.6	10.99	3.3	0.194	0.0277	0.1433	0.7551	0.6063
GPx (nmol/min·mL)	132.33	73.25	102.52	103.06	53.04	<0.0001	0.9626	<0.0001	0.0403	0.9838
SOD (U/mL)	2.83	3.14	2.85	3.11	1.15	0.1377	0.2121	0.3471	0.76	0.0172

SeE, vitamin E+selenium; ExP, plant extracts; SEM, standard error of the mean; TBARS, thiobarbituric acid reactive substances; MDA, malondialdehyde; GPx, glutathione peroxidase; SOD, superoxide dismutase.

**Table 6:** Protein levels (%) by electrophoresis in serum of Dorper×Santa Ines lambs receiving a high-concentrate diet with different antioxidant additives

Items	SeE		ExP		SEM	Pr>F		
		
	With	Without	With	Without		SeE	ExP	SeE×ExP
Haptoglobin	0.26	0.27	0.24	0.28	0.030	0.653	0.035	0.620
Ceruloplasmin	1.50	1.50	1.47	1.53	0.116	0.994	0.701	0.213
Albumin	67.39	66.46	67.19	66.66	0.666	0.335	0.583	0.536
IgG heavy-chain	10.04	10.45	10.36	10.13	0.434	0.514	0.713	0.768
Transferrin	9.00	9.11	9.24	8.87	0.242	0.713	0.216	0.729
Glucopolysaccharide	0.51	0.53	0.53	0.51	0.048	0.613	0.697	0.251
IgG light-chain	4.03	3.98	4.06	3.95	0.271	0.859	0.781	0.302

SeE, vitamin E+selenium; ExP, plant extracts; SEM, standard error of the mean; IgG, immunoglobulin G.
